# Social and non-social risk-taking in adolescence

**DOI:** 10.1038/s41598-025-90050-y

**Published:** 2025-02-26

**Authors:** Weike Wang, Kylie Evans, Susanne Schweizer

**Affiliations:** 1 University of New South Wales, Sydney, Australia; 2 University of New England, Armidale, Australia

**Keywords:** Social risk-taking, Adolescence, Depressive symptoms, Social value, Balloon analogue risk task, Human behaviour, Psychology, Risk factors

## Abstract

The Social Risk Hypothesis of Depression proposes that individuals who perceive themselves as low in value to their social groups are at risk of developing depression. Behaviourally, lower self-perceived social value is proposed to reduce individuals’ propensity to take social risks to avoid further lowering their social worth. This is in contrast with adolescent-typical behaviour, which is characterised by heightened risk-taking in social contexts. The current study aimed to investigate how low self-perceived social value influences risk-taking in social compared to non-social contexts during adolescence. 114 adolescents (aged 12–23 years) completed the Balloon Analogue Risk Task (BART) in individual and social contexts. The results showed that adolescents took more risks in social compared to individual contexts. Risk-taking across social and individual contexts also varied as a function of self-perceived social value. In older—but not younger—youth, lower self-perceived social value was associated with greater risk-taking in social compared to individual contexts. These findings suggest that self-perceived social value differentially influences social risk-taking across adolescence. In later youth, the heightened social risk-taking observed in individuals with low self-perceived social value aligns with developmental theories suggesting that risk-taking at this age serves to increase social rank and avoid social exclusion by peers.

**Supplementary Information:**

The online version contains supplementary material available at 10.1038/s41598-025-90050-y.

## Introduction

Depression, the worldwide leading cause of disability in adolescents^1^, is associated with increased real-world risk-taking^2^. However, lab-based evidence on the association between depression and risk-taking in adolescence (10–24 years)^3^ is limited and mixed. The dissociation between real-world and lab-based risk-taking may in part be due to the social contexts in which real-world risks are commonly taken. Risk-taking in adolescence has been proposed to serve the function of reducing individuals’ possibility threat potential for likelihood of susceptibility to social exclusion, to which adolescents are particularly sensitive^4^. Social sensitivity refers to individuals’ “motivation toward social relatedness” (p.121)^5^ that potentiates the attention and affective salience attributed to information concerning social evaluation by others^5,6^. Excessive social sensitivity has been associated with increased levels of depressive symptoms, especially in adolescence^7^. Understanding the interplay between adolescent depressive symptomatology and risk-taking in social versus individual contexts can inform prevention and early intervention for both depression and maladaptive risk-taking behaviours in adolescents.

### Reduced social risk-taking in depression: a social risk hypothesis perspective

A useful theoretical framework for considering risk-taking in social contexts in adolescents at risk for depression is the *Social Risk Hypothesis of Depression*^8^. The social risk hypothesis posits that depression emerges as a defensive response to temporarily minimise the risk of social exclusion due to reduced social investment potential. Social investment potential refers to the ratio of an individual’s social value (i.e., the resources provided to others due to one’s participation in the social group) relative to their social burden (i.e., the loss of current or potential resources for one's social group which are attributable to the self). When an individual’s social value and social burden approach equivalence, their self-perceived risk of social exclusion increases. In order to raise social investment potential and avoid exclusion, depression motivates individuals to adopt risk-averse strategies that forgo social gains to avoid potentially costly social losses^8^. Consequently, depression should be associated with reduced risk-taking, especially in social contexts. Empirical evidence investigating the impact of depressive symptoms on adolescent risk-taking in labs, however, is limited. We therefore first review the literature on risk-taking and depression in adulthood.

Reduced social risk-taking has been observed in adults with depression on a range of tasks, including the Balloon Analogue Risk Task (BART). On the BART^9^, participants pump up a balloon to gain points, with bigger balloons awarding more points, that are converted to monetary rewards. However, each pump increases the chance of the balloon bursting. In an elegant study that directly compared risk-taking in social versus individual contexts, Follett et al.^10^ showed that adults with depression demonstrated significantly less risk-taking than never-depressed controls only when they were completing the BART in a social setting, but not when they performed the task in an individual setting (i.e., for solely their own gain). That is, they took less risks only when the points they risked gaining or losing were counted towards a group total, not when they played for their own losses and gains. Reduced social risk-taking in depression has also been observed with other behavioural paradigms that incorporated social contexts. For example, studies using interpersonal trust-reciprocity games found that people with depression made fewer deceptive (risky) choices than healthy controls, when the risk of being detected was low^11,12^. In contrast, the relationship between depression and risk-taking was equivocal in non-social contexts, with previous research showing, negative^13,14^, positive^15,16^ and no^17,18^ associations. In other words, depression may reliably reduce risk-taking in social domains, but not necessarily other domains.

In line with the social risk hypothesis of depression, preliminary evidence also suggests a negative association between social risk-taking and one’s perceived social value. Individuals with low self-esteem were less willing to join a new social group (i.e., take a social risk) compared to those with high self-esteem, especially when acceptance by the group was ambiguous rather than certain^19^. Compared to the low self-esteem group, individuals with high self-esteem also endorsed more risky decision-making (e.g., delivering a risky joke in public) in the face of relational threat^20^. Taken together, studies conducted in adults have generally supported the social risk hypothesis of depression’s tenet that individuals with low self-perceived social value and at-risk for depressive symptoms are socially risk-averse.

### Adolescent risk-taking and the role of social value: a developmental perspective

In adolescence, sensitivity to social exclusion is heightened. The social risk hypothesis of depression argues that those with particularly low self-perceived social value may withdraw socially and take fewer social risks, thereby can withdraw socially and take fewer social risks, thereby limiting opportunities for social support and exacerbating the risk for depression. However, this prediction is in contrast with the age-typical risk-taking propensity observed in adolescents^21^ as well as meta-analytic evidence that suggests increased real-world risky behaviours in depressed adolescents^2^. Such discrepancy may be partially explained by developmental theories that highlight the function and influence of increased social sensitivity in adolescence.

Specifically, the prioritisation of social goals in adolescence may motivate rank-seeking and socially rewarding behaviours in adolescence^22^. Adolescents may engage in risk-taking to impress peers and achieve or maintain higher social status, and to avoid social exclusion^23^. Indeed, Blakemore and colleagues have suggested that when physical or health risks are pitted against social risks (i.e., the risk of being rejected by peers), adolescents overweigh social risks and are willing to engage in excessive risk-taking behaviours to avoid the risk of social rejection^4,24^. This is further supported by neuroimaging findings suggesting that adolescent risk-taking may be influenced by the desire for social-status enhancement^25^. Recruitment of the anterior insula, a region associated with reward processing and deliberation processes in risk-taking^26^, was increased in a social rank feedback condition compared to a monetary feedback condition. Similarly, a simulated social exclusion paradigm led to increased risk-taking on a monetary gambling task in the group that anticipated social exclusion, but not in control groups^27^. Relatedly, social connectedness (the opposite of social exclusion) was associated with fewer health risk behaviours^28^. Heightened social risk-taking, then, may be a rational response from adolescents to avoid social exclusion by peers^29^.

This developmental tendency towards risk-taking to limit the risk of social exclusion may be potentiated in adolescents who perceive their social value as low, especially those who experience symptoms of depression. Preliminary evidence suggests a positive association between depression and social risk-taking in adolescence. For example, depressed adolescents (15 years) took more risks than non-depressed controls in an interpersonal trust game, but not a non-social lottery game^30^. Similarly, adolescents (14–18 years) with low self-esteem (arguably, individuals that view themselves as low in social value) showed more risk-taking than adolescents with high self-esteem in social compared to non-social contexts on lab-based^31^ and self-report measures of risk-taking^32^. However, this literature is in contrast with findings using non-social risk-taking tasks that show reduced risk-taking in young people who are clinically depressed^33^, adolescents who report more emotional disorder symptoms^34^ and adolescents at-risk for depression^35^. The current study therefore aimed to determine the impact of depressive symptoms and low self-perceived social value on adolescent risk-taking across social and non-social contexts.

### The current study

To study risk-taking across social and non-social contexts the current study recruited 114 participants aged 12–23. Participants completed the BART in both a social (points gained for the group) and non-social/individual (points gained for self) condition. Age related differences were explored as risk-taking peaks around 18–19 years of age^36,37^, yet peer influence on risk-taking is highest early in adolescence^38^.

In line with the reviewed literature the study tested three hypotheses: first, risk-taking was proposed to increase with age (Hypothesis 1). Second, across adolescence risk-taking would be higher in the social compared to non-social condition (Hypothesis 2a), and this effect would be greatest early in adolescence (i.e., inverse association with age; Hypothesis 2b). Third, higher levels of depressive symptoms (Hypothesis 3a) and lower self-perceived social value (Hypothesis 3b) would be associated with more risk-taking in the social condition, especially in younger adolescents.

## Results

### Participant demographics and characteristics

Participant demographic and clinical characteristics are included in Table 1.

**Table 1:** Participant characteristics.

		M (SD)/ *N* (%)
Age Range: 12–23 years		16.23 (2.74)
Gender	Female	72 (63.16%)
	Male	40 (35.09%)
	Other	0
	Prefer not to say	2 (1.75%)
Ethnicity	Asian	46 (40.35%)
	White	33 (28.95%)
	Other	24 (21.05%)
	Aboriginal or Torres Strait Islander	3 (2.63%)
	Mixed	3 (2.63%)
	Black	2 (1.75%)
	Hispanic	0
	Prefer not to say	3 (2.63%)
Depressive symptoms		6.19 (4.68)
Self-perceived social value		60.62 (15.18)

Depressive symptoms = Depression subscale of the DASS-21^39^; Self-perceived social value = Total score on the Social Comparison Scale^40^.

### Risk-taking in social and individual contexts during adolescence

In contrast with our first hypothesis, there was no significant linear or quadratic relationship between age and risk-taking (Table 2). Given previous research suggesting that risk-taking peaks around 19 years^36^, we also examined the association between age and risk-taking by applying median split, separating participants into younger (12–17 years) and older (18–23 years) age groups. When using these age groupings, the predicted age-related increase in risk-taking emerged, with the older age group engaging in significantly more risk-taking compared to the younger age group (see Supplementary Table S3, Figure S1).

**Fig. 1:**
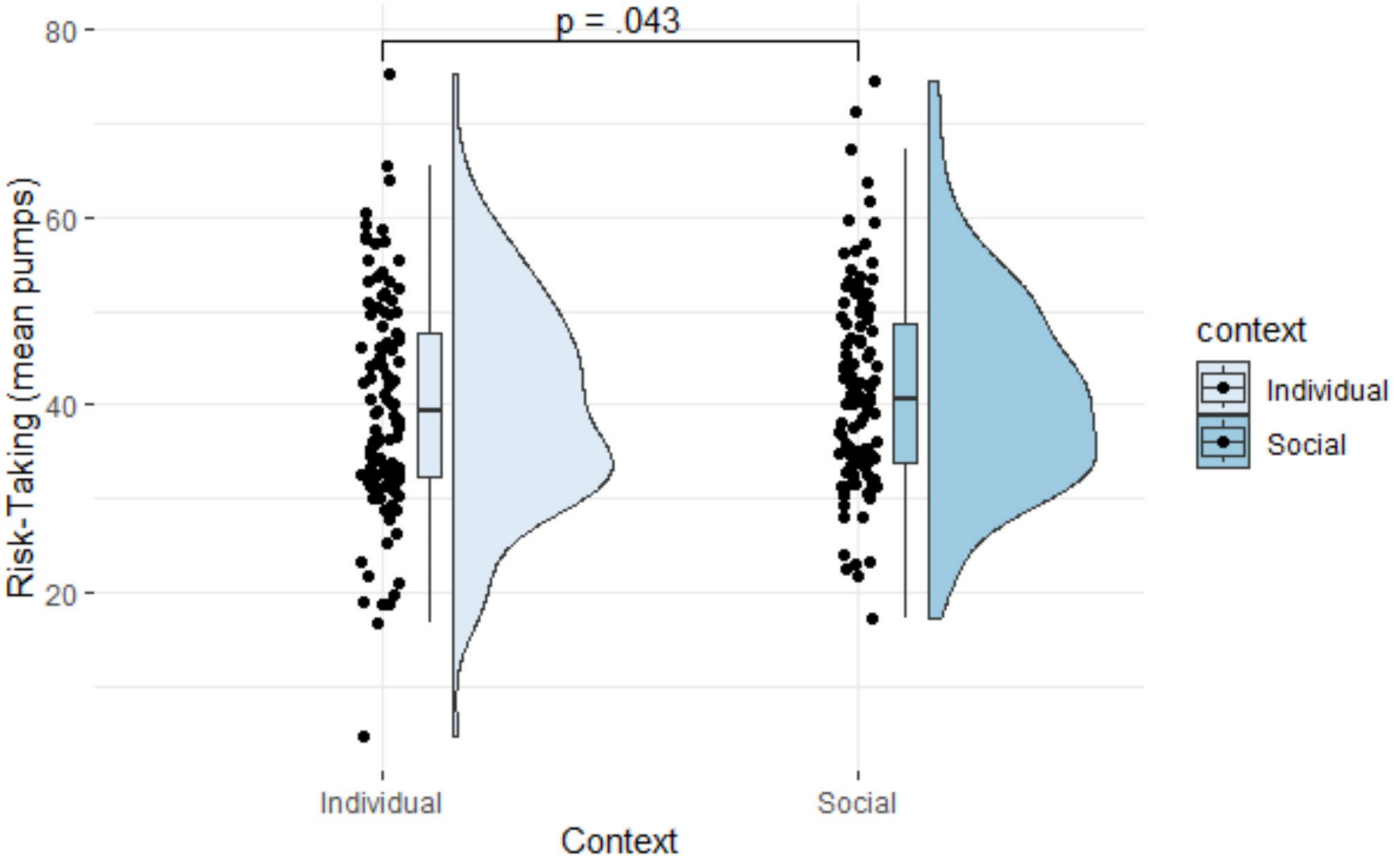
Effect of context on risk-taking measured as mean pumps. *Note.* The figure illustrates differences in mean pumps on the Balloon Analogue Risk Task in individual versus social contexts. In the individual context, participants played the Balloon Analogue Risk Task for their own gain. In the social context, they played the Balloon Analogue Risk Task to contribute towards the group’s gain.

In line with the second hypothesis, individuals across the adolescent age range took more risks in the social compared to the individual context, as evidenced by a main effect of context (Fig. 1; Table 2, Model H2a). However, age did not interact with context, suggesting that the effect of context did not vary across the adolescent age range (Table 2, Model H2b).

### Depressive Symptoms and social risk-taking in adolescence

**Table 2:** The effects of age and context on risk-taking.

	Model H2a				Model H2b			
	*β*	*SE*	CI	*p*	*β*	*SE*	CI	*p*
Linear effect of age								
(Intercept)	**40.01**	**1.03**	**37.99–42.03**	**< 0.001**	**40.01**	**1.03**	**37.99–42.03**	**< 0.001**
Age	1.43	0.97	−0.48–3.34	0.143	1.37	1.03	−0.66–3.40	0.185
Context	**1.41**	**0.69**	**0.05–2.77**	**0.043**	**1.41**	**0.69**	**0.05–2.77**	**0.043**
Age × Context					0.12	0.69	−1.24–1.48	0.861
Marginal/Conditional R^2^	0.021 / 0.780				0.021 / 0.780			
Quadratic effect of age								
(Intercept)	**40.99**	**1.40**	**38.23–43.76**	**< 0.001**	**40.78**	**1.44**	**37.93–43.62**	**< 0.001**
Age	1.68	1.00	−0.28–3.64	0.094	1.57	1.06	−0.52–3.65	0.141
Age^2^	−0.99	0.97	−2.89–0.92	0.309	−0.77	1.02	−2.79–1.25	0.454
Context	**1.41**	**0.69**	**0.05–2.77**	**0.043**	1.84	0.97	−0.07–3.75	0.060
Age × Context					0.23	0.71	−1.17–1.63	0.745
Age^2^ × Context					−0.43	0.69	−1.79–0.92	0.530
Marginal/Conditional R^2^	0.029 / 0.780				0.029 / 0.781			

Context = Social versus Individual. Individual context refers to the condition where participants play the Balloon Analogue Risk Task for their own gain. In the social context, they played the Balloon Analogue Risk Task to contribute towards the group’s gain. Age was standardised. Significant values are in bold.

Contrary to our third hypothesis (H3a), risk-taking across social and individual contexts did not differ as a function of adolescent depressive symptoms (Table 3, Model H3a). There was no main effect of depressive symptoms, suggesting that in this sample depressive symptoms were unrelated to risk-taking on the BART. Moreover, depressive symptoms did not interact with context to predict risk-taking, and the predicted three-way interaction between context, depressive symptoms, and age was not significant (Table 3, Model H3a).

**Table 3:** The effects of age and context on risk-taking across levels of depressive symptoms and self-perceived social value.

	Model H3a: Depressive Symptoms				Model H3b: Self-Perceived Social Value			
	*β*	*SE*	CI	*p*	*β*	*SE*	CI	*p*
(Intercept)	**40.09**	**1.01**	**38.09–42.09**	**< 0.001**	**39.94**	**1.02**	**37.93–41.94**	**< 0.001**
Age	1.57	1.03	−0.45–3.60	0.128	1.42	1.02	−0.59–3.44	0.166
Context	**1.43**	**0.69**	**0.08–2.79**	**0.040**	1.29	0.68	−0.05–2.62	0.060
Moderator	−0.65	1.02	−2.66–1.37	0.528	−0.08	1.05	2.15–1.99	0.940
Age × Context	0.19	0.70	−1.18–1.57	0.784	0.19	0.68	−1.15–1.53	0.781
Age × Moderator	1.67	1.13	−0.55–3.89	0.140	−0.82	1.02	−2.83–1.19	0.421
Context × Moderator	0.19	0.69	−1.17–1.55	0.784	−0.42	0.70	−1.80–0.96	0.551
Age × Context × Moderator	0.44	0.76	−1.07–1.94	0.570	**−1.43**	**0.68**	**−2.77–−0.09**	**0.037**
Marginal/Conditional R^2^	0.046 / 0.781				0.044 / 0.788			

Moderator is either standardised depressive symptoms or standardised self-perceived social value; see model names. Age refers to standardised age. Context refers to social versus individual context. In the individual context participants played the Balloon Analogue Risk Task for their own gain. In the social context, they played the Balloon Analogue Risk Task to contribute towards the group’s gain. Significant values are in bold.

### Self-perceived social value and social risk-taking in adolescence

**Fig. 2:**
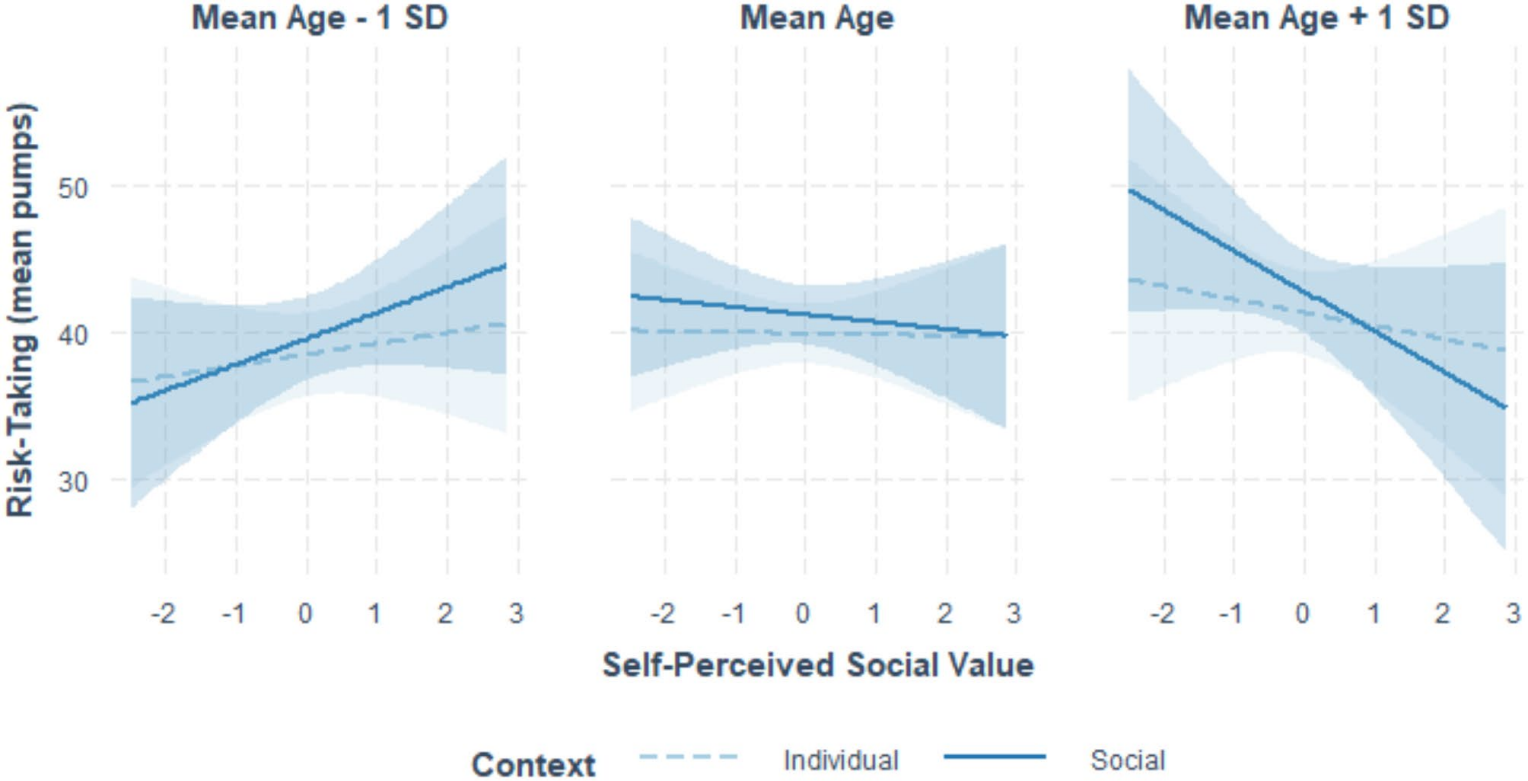
The effects of age and perceived social value on risk-taking across contexts. *Note.*The figure illustrates the effect of self-perceived value across context (social vs. individual) in early youth (left panel), middle youth (middle panel) and later youth (right panel). Self-perceived social value was operationalised as the total score on the Social Comparison Scale^40^. Self-perceived social value and age were standardised. Individual context refers to the condition where participants play the Balloon Analogue Risk Task for their own gain. In the social context, they played the Balloon Analogue Risk Task to contribute towards the group’s gain.

In line with our hypothesis (H3b), risk-taking profiles did vary across age in social compared to individual contexts as a function of self-perceived social value (Fig. 2). That is, there was a significant three-way interaction between age, social context and self-perceived social value (Table 3, Model H3b).

To break down the three-way interaction, we compared estimated marginal means between mean pumps in social and individual contexts across different levels of self-perceived social value and age (Table 4; Fig. 2). Results revealed that lower self-perceived social value (1 SD below the mean) in older youth (1 SD above mean age) was associated with greater social compared to individual risk-taking. No significant differences in social compared to individual risk-taking were observed across levels of self-perceived social value in early and middle adolescence.

**Table 4:** Estimated marginal means and effect sizes for the difference in mean pumps in social compared to individual contexts across age and self-perceived social value.

	Mean Age − 1 SD				Mean Age (0)				Mean Age + 1 SD			
	Estimated marginal mean	*SE*	*p*	Cohen’s d	Estimated marginal mean	*SE*	*p*	Cohen’s d	Estimated marginal mean	*SE*	*p*	Cohen’s d
Low self-perceived social value (−1)	0.08	1.38	0.951	0.02	1.70	0.98	0.085	0.33	**3.32**	**1.43**	**0.022**	**0.65**
Average self-perceived social value (0)	1.10	0.98	0.266	0.22	1.29	0.69	0.065	0.25	1.48	0.98	0.133	0.29
High self-perceived social value (1)	2.11	1.24	0.091	0.41	0.87	1.00	0.388	0.17	−0.37	1.51	0.806	0.07

Self-perceived social value was operationalised as the total score on the Social Comparison Scale^40^. Estimated marginal means captured the difference in mean pumps in the social compared to the individual context. Self-perceived social value and age were standardised. Significant values are in bold.

## Discussion

Adolescence is a period of heightened risk-taking propensity and increased vulnerability for depression^1,21^. Although reduced social risk-taking in depression has been shown in adults, there has been limited research investigating this relationship in adolescence. The current study examined whether adolescents’ depressive symptoms and/or self-perceived social value were associated with their risk-taking propensity in social compared to non-social contexts.

Results showed that all adolescents took more risks in the social compared to individual condition, and that this effect did not differ across levels of depressive symptoms. Low self-perceived social value, however, was associated with increased social compared to individual risk-taking, but only in older youth.

Older youth showed more overall risk-taking. While this effect only emerged when modelling age as a dichotomous (median split) rather than continuous variable, it is in line with previous research. Behavioural and neurodevelopmental evidence has shown that adolescent risk-taking and related brain activation in a network including the anterior insula and dorsal medial prefrontal cortex peaks around 19 years of age^37,41,42^.

The observed greater social, relative to individual, risk-taking supports theoretical frameworks that propose the minimisation of social risk as a key motivator for risk-taking in adolescence^4,24^. Heightened risk-taking in social compared to individual contexts is in line with previous developmental studies that introduced social context through peer observers. For example, in the Stoplight game, participants engage in a driving simulation in the presence and absence of peer observers^31,43^. The paradigm used in the current study introduced social context through collaboration towards a shared goal (i.e., accumulating sufficient points to be eligible for bonus rewards). Importantly, this paradigm involves all social agents simultaneously. This provides unique opportunities for future research to test the paradigm’s utility in establishing the impact of not only self-perceived, but also objective social rank, as the task can be completed within existing social networks such as school classes or online social networks.

The observed heightened social risk-taking could be directed to promote prosociality and wellbeing during adolescence by channelling risk-taking propensity to prosocial outlets^44,45^. For example, taking certain social risks, such as initiating a new friendship or speaking out against popular opinions, could be modelled as socially acceptable or socially rewarding in schools or communities. The experiences of positive outcomes from taking positive social risks may further increase adolescents’ tolerance of social risk, which in turn, increase prosocial tendencies and promote mental wellbeing^46^.

However, in contrast with previous findings, social risk-taking was not potentiated in younger adolescents, who have been shown to be particularly sensitive to peer influence^43^. The different operationalisation of social context in the current task-design may have reduced the potential for peer influence. Unlike most peer influence research^38,47^, there was no established behavioural norm for performance on the task that could be conformed with or violated by participants. Instead, self-perceived social value was arguably more readily captured in this task, as it provided participants with a numeric quantification of their “value” to the group.

Indeed, self-perceived social value did predict differential risk-taking across individual and social contexts. Older youth with lower self-perceived social value took more risks in the social compared to the individual context. These findings highlight different patterns of risk-taking compared to adults. That is, adults (aged over 24 years) with low self-perceived social value will avoid social risk-taking, while young people seek it out^19,20,48^. These age-related differences suggest that low perceived social value may amplify risk-taking in social contexts during a period when risk-taking propensity is at its highest. Young people low in self-perceived social value may then need extra support to help them avoid excessive risk-taking in social contexts, as they may be more motivated to engage in risky behaviours in pursuit of a rapid increase in social value.

An alternative, arguably complementary, explanation for the heightened risk-taking in older youth with low self-perceived social value is that they may conceive of themselves as having nothing to lose, further motivating their social risk-taking appetite. However, the current study cannot speak to differential loss avoidance versus reward-seeking. The heightened sensitivity to social context, which the current study observed in youth with low self-perceived social value, can potentially can potentially be harnessed for good. If peer-groups model psychologically healthy behaviours, such as good sleep and digital hygiene, youth with low self-perceived social value may perceive it as risky not to conform with these group norms. Engaging in these wellbeing promoting activities can in turn confer preventative advantages for these at-risk individuals.

In contrast with the effect of self-perceived social value on risk-taking, the current study showed no effect of depressive symptoms on risk-taking in adolescents. This aligns with findings from the study using this paradigm in adults^10^. The differential effect of social context only emerged when comparing clinically depressed individuals to never depressed adults. Indeed, the significant group by context interaction remained significant after they controlled for individual differences in depressive symptoms. Similarly, other adult studies that showed reduced social risk-taking were all conducted with clinically depressed participants^11,12,49^. Future research should therefore compare social to non-social risk-taking in clinically depressed adolescents.

Another potential reason for the lack of an association between adolescents’ depressive symptoms and overall risk-taking, and social risk-taking in particular, could be that cognitive biases associated with depression become more entrenched in adulthood when they exert a greater effect on behaviour. In line with this account, depressogenic cognitive biases increase across adolescence^50^ and heightened risk-taking in depression marginally increases with age^51^. However, some previous studies have also failed to show an association between depression and risk-taking, even in clinical samples for both adults^17,18^ and adolescents^52,53^. Additionally, while others showed reduced risk-taking on standard individual lab tasks^34^ or greater social risk-taking^30^ with elevated emotional symptoms, they either only investigated risk-taking in non-social contexts or did not compare social to individual risk-taking. The current study adds to this mixed body of evidence in an important way, as it directly compares social versus individual risk-taking on the same task. To further elucidate the potential association between adolescent depression and risk-taking, future research should systematically compare social to individual risk-taking.

The results from the current study should be interpreted within the context of several limitations. First, while the BART is a widely adopted behavioural measure for risk-taking, the risk-taking propensity being measured may actually change throughout the task as people learn about the probability of balloon bursts^54^. Future research is needed to adapt other behavioural measures of risk-taking that disentangle risk preference and expected value (e.g., the Columbia Card Task^55^) to social contexts. Second, behavioural measures (e.g., adjusted mean pumps, total points, total bursts) of risk-taking on the BART fail to capture variance of trial-level pumps. Novel analyses such as computational modelling (e.g., Exponential-Weighting Mean-Variance model in Park et al.^56^) should be considered in future studies to investigate risk-taking propensity measured by the BART. Third, as noted above, the current study cannot speak to the impact of clinical depression on social risk-taking in adolescence, though the reviewed evidence suggests this may be heightened. Fourth, although adequately powered, the current study included a relatively small sample of adolescents. The observed age-related differences should not be interpreted as developmental changes, as these need to be confirmed by future longitudinal research. Future studies should include a larger sample and a wider age range collected across time to be able to draw developmental inferences. Finally, social groups in this experiment were randomly created by the experimenters, and thus studying risk-taking in real-world groupings is a critical next step.

In summary, adolescents appeared to take more risks in social compared to non-social contexts. Although we did not find an association between depressive symptoms and social risk-taking, we did find that self-perceived social value differentially influenced social risk-taking across adolescence. The findings suggested that social risk-taking peaked in individuals with the lowest self-perceived social value during later youth. Social risk-taking in this developmental period, we argue, may be a rational response to the threat of low social investment potential and risk of social exclusion.

## Method

### Participants and procedures

The study was approved by the University of New South Wales Human Research Ethics Executive Committee (HC number 3643) and Human Research Ethics Advisory Panel C (HC220267). The experiment was performed in accordance with relevant regulations and the Declaration of Helsinki. Based on power calculations using G*Power 3.1^57^, a sample size of *N* = 102 was required to achieve a power of 90% to detect an effect of age group and depression on risk-taking across social versus non-social conditions. The sample size was calculated assuming a moderate effect size of *η*_*p*_^2^= 0.15^10^ with significance threshold at *α* = 0.05. We note that the sample size was calculated with age initially treated as a two-level categorical variable. Following a reviewer’s suggestion, we reanalysed the data using age as a continuous variable and reported these results. One-hundred-and-fourteen participants were recruited. Participants were 63 school students (*M*_age_ = 14.06 years, *SD*_age_ = 1.23, 42 Female, 20 Male, 1 Prefer not to say) and 51 undergraduate students (*M*_age_ = 18.90 years, *SD*_age_ = 1.36, 30 Female, 20 Male, 1 Prefer not to say). The school-based participants were volunteers recruited through a regional high school in Australia and received an AU$20 Prezzee voucher for participation. The university-based participants were recruited from the University of New South Wales and received course credit for participation. In addition to the base compensation, participants had the opportunity to win up to an extra AU$5 depending on task performance.

All participants provided informed consent and for participants under 18 years informed consent from parents/guardians was also obtained. Participants then completed questionnaires before completing the BART twice, once in the social and once in the individual (non-social) condition. The presentation order of the conditions was counterbalanced across participants. The study was administered in person and presented on Gorilla (https://gorilla.sc/).

### Measures

#### Depressive symptoms

Symptoms of depression were measured using the 7-item depression subscale of the short version of the Depression, Anxiety and Stress Scale (DASS-21)^39^. Participants indicated the extent to which they endorsed each item on a four-point Likert scale ranging from 0 (never) to 3 (almost always). The DASS-21 has good convergent validity and internal consistency^58^. In the current sample, the depression subscale had acceptable internal consistency, ω = 0.88.

#### Social value

Self-perceived social value was operationalised as total score on the Social Comparison Scale^40^. This measure is comprised of 11 items assessing self-perceived attractiveness, social rank and social group fit. Items are all prefaced with “In relation to others I feel” (e.g., unlikeable/likeable; or incompetent/more competent). The Social Comparison Scale has good internal consistency and factor structures^59^. In the current sample the scale had good internal consistency, ω = 0.90.

#### Risk-taking

Behavioural risk-taking was measured with the BART^9^ (see description below). The BART has demonstrated good test-retest reliability and convergent validity^60,61^. In accordance with the original BART^9^, the mean and median burst points for the balloons was set to 64.5 (range 1–128), such that half the balloons would burst at 64 pumps or fewer while the other half would burst after 64 pumps. Risk-taking was operationalised as mean pumps of the unburst balloons (i.e., total pumps made divided by the number of unburst balloons).

In the individual condition participants played for themselves whereas in the social condition participants played for the team (2–5 participants). Participants were awarded AU$2.5 if they collected 7500 points or more in the individual condition. In the social condition, points were pooled for teams of 2–5 participants. Each participant was awarded AU$2.5 if the team score reached or exceeded 7500*n points (*n* = number of team members). In each condition, participants were presented with 30 trials. In each trial, they had the opportunity to inflate a balloon as much as they liked before banking their points. At the beginning of the trial, participants saw a screen indicating the balloon number currently being played (e.g., “Balloon 4 of 30”), as well as a running total of their points for the task overall: “So far, you’ve got [points total] points for YOURSELF/TEAM” (yourself = individual condition; team = social condition). The participants then used a mouse to click a button (“Air”) to inflate the balloon which visibly increased in size. Each click is worth 10 points. With each “Air” press, the points total for that balloon was updated. Points could be secured for each balloon by clicking on the “Collect Points” button and this would add the points from that balloon to the overall point tally. If the balloon burst before the points were banked, participants were instructed that they did not win any points for that balloon.

Mean pumps of unburst balloons in the first 10, second 10, and last 10 balloons were calculated to check the internal consistency of BART. Using confirmatory factor analysis, the internal consistencies for risk-taking in individual and social conditions were ω = 0.86 and ω = 0.78 respectively.

### Analyses

Linear mixed models were used for hypothesis testing. All mixed models included participant ID as random effect. For H1, age was included as fixed effect. The model testing H2a further included context as fixed effect and the interaction between age and context was added to the model to test H2b. Depressive symptoms (H3a) and self-perceived social value (H3b) were added to the model specified under H2b to test the third hypotheses. Analyses were conducted using the *lme4*^62^, *lmerTest*^63^ and *emmeans*^64^ packages in R (version 4.3.1)^65^. Significance threshold was set as *p* *≤* .05. Figure 1 and Figure S1 were produced using the *ggplot2*^66^, *ggrain*^67^ and *ggsignif*^68^ packages in R; Fig. 2 was produced using the *interactions *package^69^ in R.

## Electronic supplementary material

Below is the link to the electronic supplementary material.

Supplementary Material 1

## Data Availability

All data used and analysed during this study are publicly available on the Open Science Framework (https://osf.io/dhufj/).
