# Supplementary material for: Social and non-social risk-taking in adolescence
Source: Sci Rep. 2025 Feb 26;15:6880. doi: 10.1038/s41598-025-90050-y (PMC11865566; doi:10.1038/s41598-025-90050-y)
Supplement: Supplementary file 1 — Supplementary Material 1 [file 41598_2025_90050_MOESM1_ESM.docx]

**Social and Non-Social Risk-Taking in Adolescence**

Weike Wang^1^, Kylie Evans^2^, Susanne Schweizer^1*^

*^1^ University of New South Wales, Sydney, Australia*

*^2^University of New England, Armidale, Australia*

*Corresponding author: s.schweizer@unsw.edu.au

**Supplementary Information**

**Correlation Matrix**

**Table S1**

*Correlation Matrix of Measures of Interests*

|  | Age | Depressive Symptoms | Self-Perceived Social Value | Individual Risk-Taking | Social Risk-Taking |
| --- | --- | --- | --- | --- | --- |
| Age | 1.00 | -.05 | -.09* | .12 | .14 |
| Depressive Symptoms |  | 1.00 | -.39*** | -.05 | -.03 |
| Self-Perceived Social Value |  |  | 1.00 | .00 | .01 |
| Individual Risk-Taking |  |  |  | 1.00 | .78*** |
| Social Risk-Taking |  |  |  |  | 1.00 |

*Note*. Depressive symptoms = Depression subscale of the DASS*-*21^1^; Self-perceived social value = Total score on the Social Comparison Scale^2^; Individual Risk-Taking = Adjusted mean pumps in BART in individual context; Social Risk-Taking = Adjusted mean pumps in BART in social context.

**Age as Categoric Variable Analyses**

Given previous research suggesting that risk-taking peaks around 19 years^3^ we also examined the association between age and risk-taking after by applying median split separating participants into younger (12-17 years) and older (18-23 years) age groups. Participant demographic and clinical characteristics by age group are included in Table S2.

**Table S2**

*Participant Characteristics*

|  |  | All | Younger Group (12-17 years, N = 67) | Older Group (18-23 years, N = 47) |
| --- | --- | --- | --- | --- |
|  |  | M (SD)/ N (%) | M (SD)/ N (%) | M (SD)/ N (%) |
| Age |  | 16.23 (2.74) | 14.24 (1.38) | 19.06 (1.29) |
| Gender | Female | 72 (63.16%) | 44 (38.60%) | 28 (24.56%) |
|  | Male | 40 (35.09%) | 22 (19.30%) | 18 (15.79%) |
|  | Other | 0 | 0 | 0 |
|  | Prefer not to say | 2 (1.75%) | 1 (0.88%) | 1 (0.88%) |
| Ethnicity | White | 33 (28.95%) | 28 (24.56%) | 5 (4.39%) |
|  | Asian | 46 (40.35%) | 11 (9.65%) | 35 (30.70%) |
|  | Black | 2 (1.75%) | 1 (0.88%) | 1 (0.88%) |
|  | Hispanic | 0 | 0 | 0 |
|  | Mixed | 3 (2.63%) | 1 (0.88%) | 2 (1.75%) |
|  | Other | 24 (21.05%) | 21 (18.42%) | 3 (2.63%) |
|  | Aboriginal or Torres Strait Islander | 3 (2.63%) | 2 (1.75%) | 1 (0.88%) |
|  | Prefer not to say | 3 (2.63%) | 3 (2.63%) | 0 |
| Depressive symptoms |  | 6.19 (4.68) | 6.58 (4.59) | 5.64 (4.78) |
| Self-perceived social value |  | 60.62 (15.18) | 61.30 (16.61) | 59.66 (12.99) |

*Note.* Depressive symptoms = Depression subscale of the DASS*-*21^1^; Self-perceived social value = Total score on the Social Comparison Scale^2^.

When using these age groupings, the predicted age-related increase in risk-taking emerged, with the older age group engaging in significantly more risk-taking compared to the younger age group (Table S3, Figure S1).

**Table S3**

*Effects of Age Group and Context on Risk-Taking*

|  | Model H2a | | | | Model H2b | | | |
| --- | --- | --- | --- | --- | --- | --- | --- | --- |
| *Predictors* | *b* | *SE* | CI | *p* | *b* | *SE* | CI | *p* |
| (Intercept) | **38.27** | **1.29** | **35.72 – 40.82** | **<.001** | **38.14** | **1.33** | **35.53 – 40.76** | **<.001** |
| Age Group | **4.22** | **1.94** | **0.39 – 8.05** | **.032** | **4.52** | **2.07** | **0.45 – 8.59** | **.030** |
| Context | **1.41** | **0.69** | **0.05 – 2.77** | **.043** | 1.66 | 0.90 | -0.11 – 3.43 | .067 |
| Age Group × Context |  |  |  |  | -0.60 | 1.40 | -3.35 – 2.15 | .668 |
| Marginal/Conditional R^2^ | 0.039 / 0.780 | | | | 0.040 / 0.780 | | | |

*Note*. Context = Social versus Individual. Individual context refers to the condition where participants play the Balloon Analogue Risk Task for their own gain. In the social context, they played the Balloon Analogue Risk Task to contribute towards the group’s gain.

**Figure S1**

*Effect of Age Group on Risk-Taking Measured as Mean Pumps
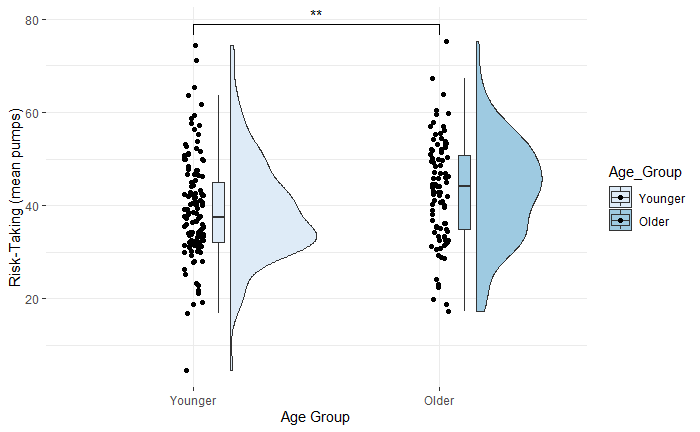
*

**References**

1. Lovibond, P. F. & Lovibond, S. H. The structure of negative emotional states: Comparison of the Depression Anxiety Stress Scales (DASS) with the Beck Depression and Anxiety Inventories. *Behaviour Research and Therapy* **33**, 335–343 (1995).

2. Allan, S. & Gilbert, P. A social comparison scale: Psychometric properties and relationship to psychopathology. *Personality and Individual Differences* **19**, 293–299 (1995).

3. Duell, N. *et al.* Age Patterns in Risk Taking Across the World. *J Youth Adolescence* **47**, 1052–1072 (2018).
